# Sensitivity and specificity of Varian Halcyon's portal dosimetry for plan‐specific pre‐treatment QA

**DOI:** 10.1002/acm2.14001

**Published:** 2023-04-22

**Authors:** Gary Razinskas, Robert Schindhelm, Otto A. Sauer, Sonja Wegener

**Affiliations:** ^1^ Department of Radiation Oncology University Hospital Wurzburg Wurzburg Germany

**Keywords:** error detection sensitivity and specificity, plan‐specific quality assurance, portal dosimetry

## Abstract

**Purpose:**

Developed as a plan‐specific pre‐treatment QA tool, Varian portal dosimetry promises a fast, high‐resolution, and integrated QA solution. In this study, the agreement between predicted fluence and measured cumulative portal dose was determined for the first 140 patient plans at our Halcyon linear accelerator. Furthermore, the capability of portal dosimetry to detect incorrect plan delivery was compared to that of a common QA phantom. Finally, tolerance criteria for verification of VMAT plan delivery with Varian portal dosimetry were derived.

**Methods:**

All patient plans and the corresponding verification plans were generated within the Eclipse treatment planning system. Four representative plans of different treatment sites (prostate, prostate with lymphatic drainage, rectum, and head & neck) were intentionally altered to model incorrect plan delivery. Investigated errors included both systematic and random errors. Gamma analysis was conducted on both portal dose (criteria γ_2%/2 mm_, γ_2%/1 mm_, and γ_1%/1 mm_) and ArcCHECK measurements (criteria γ_3%/3 mm_, γ_3%/2 mm_, and γ_2%/2 mm_) with a 10% low‐dose threshold. Performance assessment of various acceptance criteria for plan‐specific treatment QA utilized receiver operating characteristic (ROC) analysis.

**Results:**

Predicted and acquired portal dosimetry fluences demonstrated a high agreement evident by average gamma passing rates for the clinical patient plans of 99.90%, 96.64%, and 91.87% for γ_2%/2 mm_, γ_2%/1 mm_, and γ_1%/1 mm_, respectively. The ROC analysis demonstrated a very high capability of detecting erroneous plan delivery for portal dosimetry (area under curve (AUC) > 0.98) and in this regard outperforms QA with the ArcCHECK phantom (AUC ≈ 0.82). With the suggested optimum decision thresholds excellent sensitivity and specificity is simultaneously possible.

**Conclusions:**

Owing to the high achievable spatial resolution, portal dosimetry at the Halcyon can reliably be deployed as plan‐specific pre‐treatment QA tool to screen for errors. It is recommended to support the fluence integrated portal dosimetry QA by independent phantom‐based measurements of a random sample survey of treatment plans.

## 1 INTRODUCTION

Over the past decade modulated techniques, in particular the volumetric modulated arc therapy (VMAT),^1^ have become the standard modality in modern radiotherapy to deliver highly conformal dose distributions. The ability to achieve a superior coverage of target volumes of any complex shape while at the same time sparing organs at risk promises improved treatment outcomes for a wide range of indications.^2^, ^3^ The required photon fluence modulation is accomplished by the simultaneous modulation of aperture, gantry speed, and dose rate. Despite the obvious benefits of VMAT, the increased treatment planning and delivery complexity inevitably poses significant challenges for the accurate commissioning of the treatment planning system (TPS)^4^, ^5^ as well as for plan‐specific quality assurance (QA)^6^ as prerequisites for patient safety and fidelity of treatment.

The QA of modulated treatment plans is routinely conducted by measuring dose to a phantom once before the actual treatment starts.^6^ Initially, film dosimetry was extensively adopted for this task due to appealing properties such as high spatial resolution, energy and dose rate independence, and due to the lack of practical alternatives.^7^, ^8^ Today, commercialized phantoms, containing detector arrays, dedicated to this verification task are readily available.^9^, ^10^

Aiming at reducing the burden of time‐consuming phantom setup impacting departmental resources, interest in using electronic portal imaging devices (EPIDs) attached to the linac for dosimetric verification in pre‐treatment as well as in‐vivo QA measurements has significantly grown.^11^, ^12^, ^13^, ^14^ Appealing properties of EPIDs include the high achievable contrast, large sensitive area, pixel density, and the linear response to radiation dose.^15^, ^16^

Meanwhile, increasing efforts are being made to confirm accuracy of dose delivery and to detect errors during daily treatment. Monitoring every treatment fraction in real‐time is feasible by means of machine‐parameter log file analysis,^17^, ^18^ or a transmission detector directly placed in front of the linear accelerator (linac) head^19^, ^20^, ^21^, ^22^ or EPIDs. The latter proved capable of detecting differences in beam delivery, patient position, and patient anatomy.^23^

In 2017 a fast‐rotating, O‐ring mounted delivery system—the Halcyon linac (Varian Medical Systems, Palo Alto, CA)—was introduced with a workflow focused on high patient‐throughput and intensity‐modulated treatments. It is equipped with an amorphous silicon (a‐Si) photodiode based EPID, which is always in‐line with the beam and designed for the daily check of the machine performance and as tool for plan‐specific pre‐treatment QA. Moreover, transit images are acquired automatically for each treatment field and made available in ARIA for visual inspection. Available evaluation tools allow for a mere constancy check between fractions. The implementation of additional software tools for in vivo dosimetry or the use of third‐party software would also allow for “every patient, every monitor unit” QA.^24^

Since its introduction, the Halcyon platform proved capable of delivering treatment plans of comparable quality to conventional C‐arm linacs for a multitude of different treatment sites,^25^, ^26^, ^27^, ^28^, ^29^, ^30^, ^31^, ^32^ with high mechanical leaf positioning accuracy.^33^ De Roover et al.^34^ reported on the comprehensive validation of the preconfigured Halcyon beam model and the quality of the achieved treatment delivery with multiple detectors including portal dosimetry. The feasibility of in‐vivo portal dosimetry was also demonstrated.^35^

The presented study aims at validating the ability to detect deviations between planned and delivered fluences for VMAT and IMRT plans by portal dosimetry measurements acquired at the Halcyon linac. In addition, understanding the system's sensitivity to detect potentially relevant inaccuracies in treatment plan delivery is mandatory for its introduction into clinical routine. With this aim, unmodified VMAT plans spanning the complete clinical spectrum of treatment sites together with a representative set of treatment plans, in which different kinds of intentional errors were built in, were measured with both the portal imager and the ArcCHECK phantom (Sun Nuclear Corporation, Melbourne, FL),^10^ a common means for dosimetric pre‐treatment QA as reference.

While usually the equipment in different clinics differs greatly regarding treatment units and measurement devices, the Halcyon system introduces a worldwide consistency across clinics. This makes a direct inter‐clinic comparison feasible and allows other users to adhere to the presented results as a benchmark.

## 2 METHODS AND MATERIALS

### 2.1 Radiation unit

Varian's ring‐shaped Halcyon linac (Varian Medical Systems, Palo Alto, CA, version 2.0) is designed for image‐guided radiation therapy (IGRT). The treatment unit is equipped with a double stack multi‐leaf collimator (SX2 MLC) with individual leaf widths of 1.0 cm when projected at isocenter. Both layers, which possess a relative offset of 0.5 cm, can independently contribute to beam modulation. The preconfigured system supports only a single flattening‐filter‐free (FFF) beam at nominal beam energy of 6MV. For fluence measurement the Halcyon linac is equipped with a light sensitive a‐Si photodiode based EPID (aS1200, Varian Medical Systems, Palo Alto, CA). Its active area covers 43 × 43 cm^2^ with a 154 cm source‐to‐imager distance (SID) and a 1280 × 1280 pixel matrix, translating into an imaging area of 28 × 28 cm² and a resolution of about 45 pixels/cm at isoplane.

### 2.2 Portal dosimetry

Varian portal dosimetry (PD) is designed as a plan‐specific pre‐treatment QA tool for IMRT and VMAT delivery. The fluence prediction for the 6MV FFF beams of the Halcyon is handled by the Eclipse (Varian Medical Systems, Palo Alto, CA, version 15.6) TPS based on the Anisotropic Analytical Algorithm (AAA, version 15.6.06). Using an internal detector model, the detector's dose response is predicted by calculation of scatter kernels in the scintillator material of the imager. Beam‐wise fluence acquisition was done at the Halcyon linac in a setting with neither patient nor phantom in the beam during the acquisition process. Afterwards, the 2D dose matrix of each individual intensity‐modulated field is compared against the dose prediction for the identical setting. Note that the dose matrix is an overlay consisting of all fluence maps of a VMAT arc. As the system is fully integrated into the ARIA/Eclipse environment all data is stored in the ARIA oncology information system database and the dose image comparison and Gamma evaluation is done within the integrated Portal Dosimetry application.

### 2.3 Studied clinical treatment plans

The suitability of the PD unit for plan‐specific pre‐treatment QA was evaluated on the first 140 clinically applied VMAT plans after linac installation and approval for clinical treatment. This ensured coverage of a wide range of anatomical sites and target volume complexity and size. All treatment plans were generated within the Eclipse TPS with the standardized preconfigured 6MV FFF Halcyon beam model (Acuros External Beam Algorithm 15.6.06). The plans utilized one to four arcs depending on the target volume complexity and organ at risk proximity.

### 2.4 Treatment plan modifications

**FIGURE 1:**
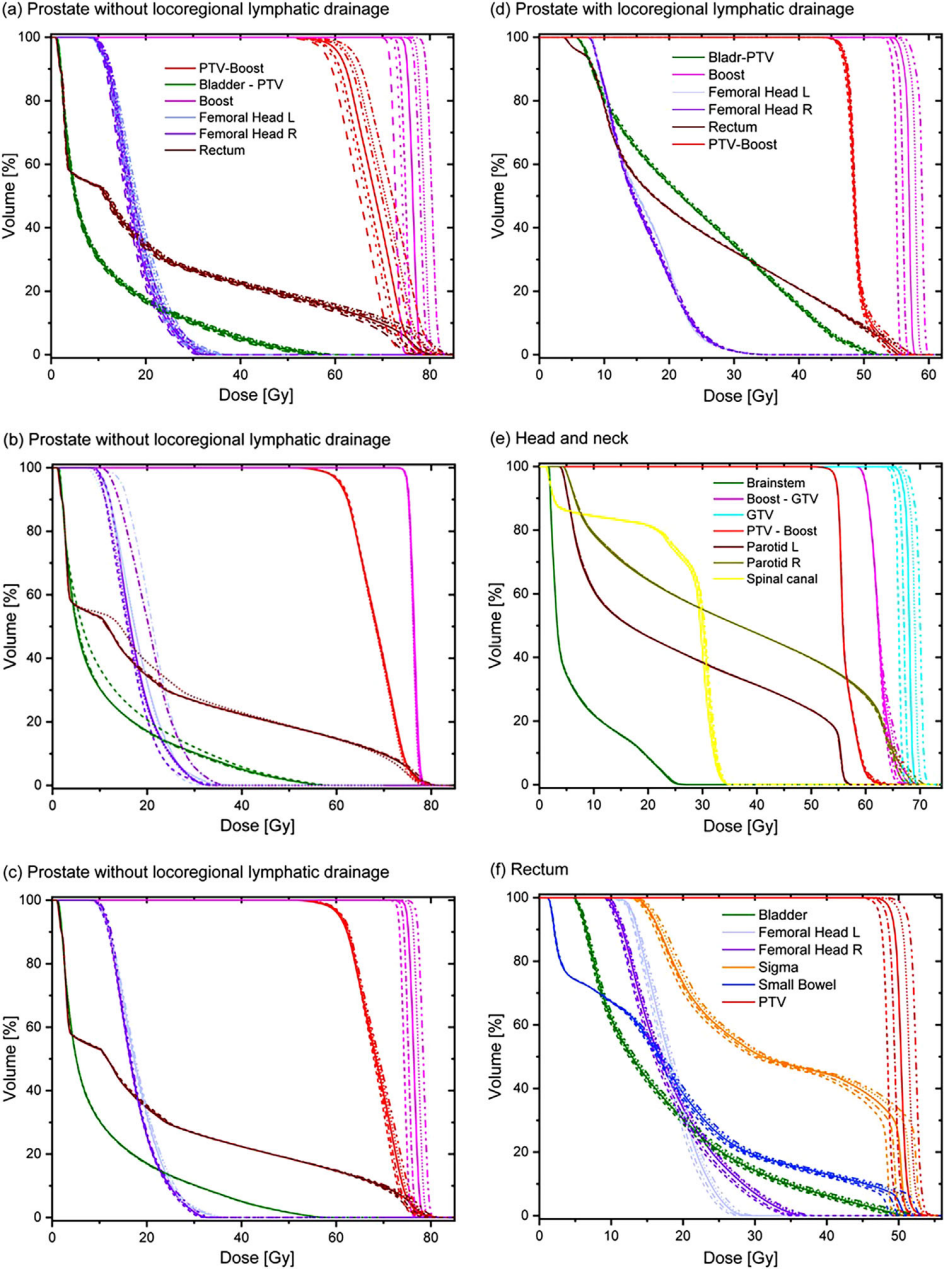
DVH overview of selected target volumes along with OARs for the clinical (solid lines) and modified (dashed lines) plan for the four treatment sites under investigation: A prostate plan without locoregional lymphatic drainage (a–c), a prostate plan with locoregional lymphatic drainage (d), a head and neck plan (e), and a rectum plan (f). The displayed errors resemble systematic MU miscalibration (a), different OAR protection (b), and altered mean dose to the high‐risk target structure (c–f).

The ability to detect relevant errors in beam delivery is imperative for the clinical use of PD for plan‐specific QA. Therefore, we investigated the basic capability of detecting both systematic and random errors with a focus on the latter. Four plans of typical treatment sites (prostate with and without the locoregional lymphatic drainage, head and neck, and rectum), in the following referred to as original plans, were selected to deliberately introduce errors. A pure scaling of the original plans’ total number of monitor units by 2%–10% simulated systematic errors (Figure 1a). The more complex random errors were achieved by a further re‐optimization of the original plans within the TPS. In the course of the optimization, the algorithm proceeds through four multi‐resolution levels progressively increasing the number of dose calculation segments and thus the accuracy of the dose calculation.

To ensure that the modified plans were not only comparable to the original plans in terms of dosimetry, but also in terms of the actual delivery process, therefore mimicking subtle changes to the exact MLC positioning, re‐optimization was performed in a “warm start” fashion. Re‐optimization commenced in either multi‐resolution level MR3 (allowing bigger adjustments to the leaf sequence) or MR4 with moderately adjusted dose levels of selected target or organ at risk (OAR) objectives, resulting in plan alternatives with slightly different dose‐volume histogram (DVH) metrics compared to their respective original plan (Figure 1b–f). DVH differences in the investigated set of 70 error‐induced plan alternatives (12–20 per original plan) include an adapted protection of OARs (changes in the mean or maximum dose of several Gy; Figure 1b) and changes in mean dose to the high‐risk target volume (1−2 Gy accumulated over all treatment fractions; Figure 1c–f). While clinical effects were the main focus in the strategy to introduce deliberate plan errors, physical effects of the studied plan perturbations manifest in changes in leaf positions and segment MU weights. The mean absolute deviation in segment MU per treatment field was 3.4%.

### 2.5 Analysis of portal dosimetry QA

All treatment plans including the error plans were delivered on the portal imager. The fluence map recorded by the imager is the overlay of all fluences from all gantry angles of each treatment field integrated on top of each other. Thus, any gantry angle dependent information about the delivered dose is discarded. Subsequent gamma analysis was conducted within the Portal Dosimetry Application for each treatment field separately combining two absolute dose difference criteria, 2% and 1%, as well as two distance‐to‐agreement criteria, 2 and 1 mm, thus yielding the three criteria 2%/2 mm, 2%/1 mm, and 1%/1 mm. The low‐dose threshold was set to 10% and no realignment between predicted and measured dose maps was allowed.

### 2.6 Phantom QA

Additionally, the complete set of plans was independently delivered to the ArcCHECK phantom, an acrylic cylindrical phantom with 1386 diode detectors arranged in a helix, with the cavity plug inserted (Sun Nuclear Corporation, Melbourne, FL). Measurements of the ArcCHECK's diode doses were evaluated for the entire treatment plan using the designated software SNC patient version 6.7.3 (Sun Nuclear Corporation, Melbourne, FL). All measurement corrections (i.e., angular, heterogeneity, and field size) as well as the measurement uncertainty option for analysis were turned on. Possible setup errors were accounted for by performing auto‐shifts of up to 1 mm in each direction. Again applying a dose cutoff at 10% of the maximum measured dose global gamma passing rates were obtained for a 3%/3 mm, 3%/2 mm, and 2%/2 mm criterion.

### 2.7 Receiver operating characteristic analysis

Receiver operating characteristic (ROC) analysis is used to assess and compare the performance of various treatment plan‐specific QA evaluation criteria. This methodology, while initially utilized for quantification of tests in diagnostic imaging, had proven valuable to routinely uncover capabilities and limitations of error detection in IMRT^36^ and VMAT QA.^37^ Based on the dedicated ROC analysis tool, as provided by OriginPro v.9.0 (OriginLab, Northampton, MA), sensitivity and specificity for both PD and ArcCHECK QA using the aforementioned assessment at varying decision thresholds were evaluated. The area under the ROC curve (AUC), in the ideal situation approaching a value of 1, served as performance measure of the studied criteria. In addition, the optimum decision threshold values can be derived from the point in ROC space closest to (0,1), where both sensitivity and specificity reach their ideal value of 1.

## 3 RESULTS

### 3.1 Fluence prediction accuracy

**FIGURE 2:**
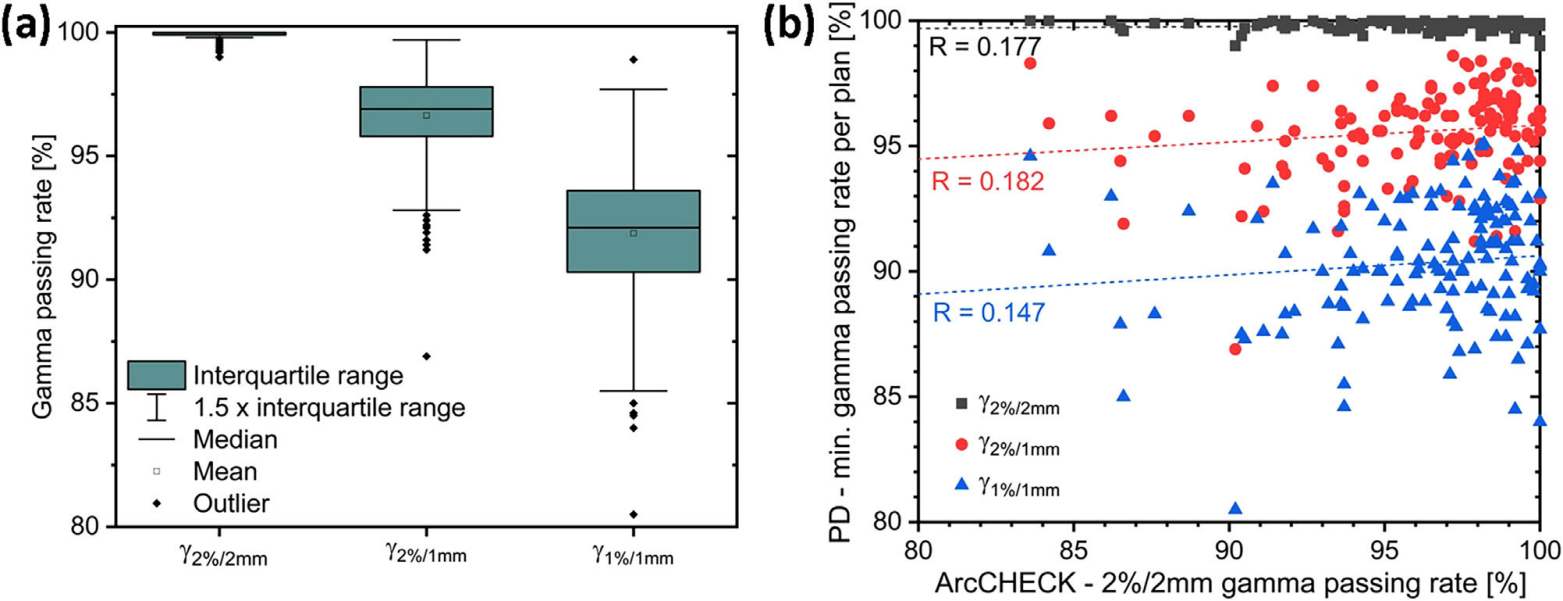
(a) Box‐plot diagram of the gamma passing rates obtained for the analysis of measured and predicted PD of all individual arcs of the investigated treatment plans. (b) Minimum PD gamma passing rates per plan versus γ_2%/2 mm_ as measured using the ArcCHECK phantom.

In terms of global gamma analysis, the measured and predicted PD images show good agreement with only a few outliers (Figure 2a). Average deviations and corresponding standard deviations across all arcs are presented in Table 1, showing a mean γ_2%/2 mm_ over all 377 VMAT arcs of 99.90%. No significant site‐specific differences in the degree of agreement were observed. The same patient plans were additionally delivered to the ArcCHECK phantom. Data for γ_2%/2 mm_ are shown in Figure 2b. There is no correlation between PD and ArcCHECK gamma passing rates (see stated values for R in Figure 2b).

**TABLE 1:** Analysis of PD and ArcCHECK plan‐specific QA at the Halcyon linac

	**Portal dosimetry**			**ArcCHECK**		
QA criterion	γ_2%/2_ _mm_	γ_2%/1_ _mm_	γ_1%/1_ _mm_	γ_3%/3_ _mm_	γ_3%/2_ _mm_	γ_2%/2_ _mm_
Total average [%]	99.90	96.64	91.87	99.34	98.62	95.81
Standard deviation [%]	0.15	1.67	2.56	1.58	2.55	4.72

Besides the total average gamma passing rate for all studied Gamma criteria the standard deviation is supplied. All stated values represent absolute and global gamma passing rates (10% low dose threshold).

### 3.2 Sensitivity of identifying plans with modified DVH metrics

**FIGURE 3:**
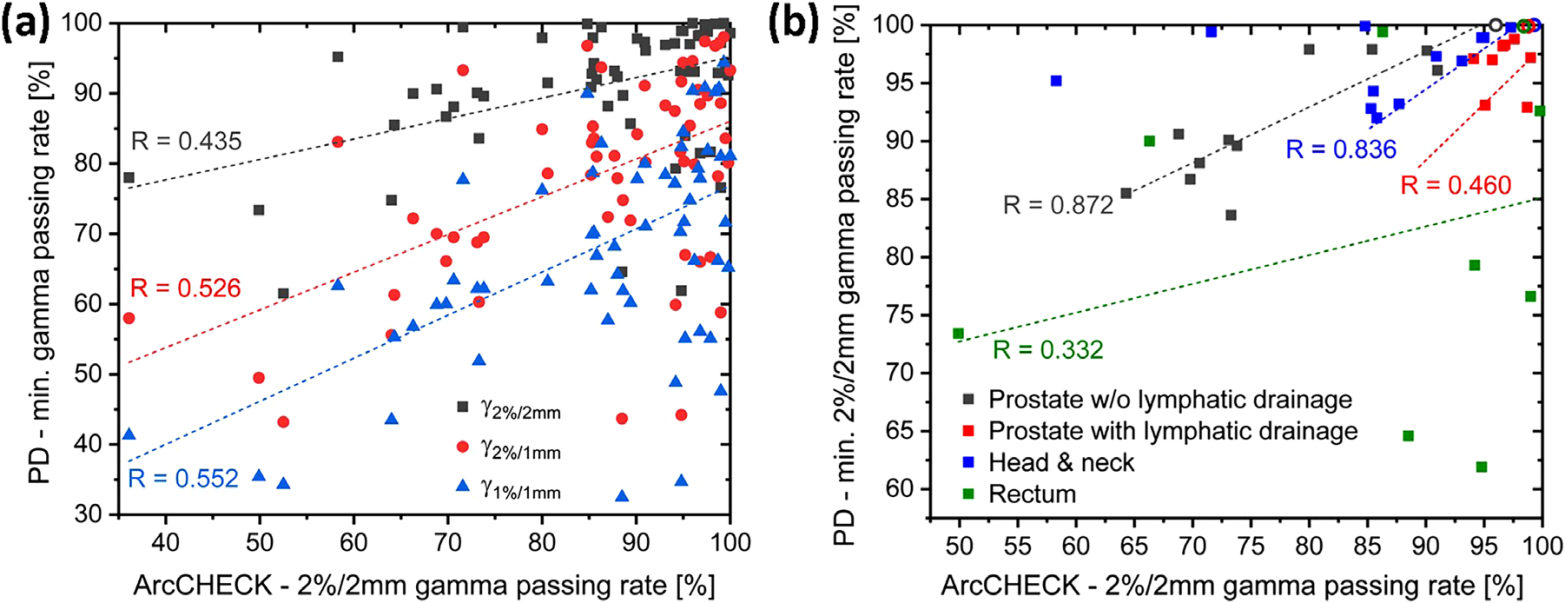
Minimum PD gamma passing rates per plan versus γ_2%/2 mm_ as measured using the ArcCHECK phantom for the re‐optimized plans. The predicted portal dose and diode signals of the respective reference plan were used to extract both quantities. (a) Overview over different gamma criteria for PD. (b) Comparison of γ_2%/2 mm_ for PD and ArcCHECK of different entities. Results are depicted for the four treatment sites under investigation. Open symbols indicate results for original plans, filled symbols indicate results for altered plans.

Analogously to the clinical plans, the modified treatment plans obtained via re‐optimizing four selected clinical plans of typical treatment sites, were evaluated. Contrary to the clinical plans, a moderate correlation between the gamma passing rates from PD and ArcCHECK measurements was observed for the altered plan variants; see Figure 3a and the correlation coefficients given therein. Naturally, all gamma passing rates are incapable of predicting how the DVH metrics changed. While for prostate without the lymphatic drainage and for the head and neck plans the correlation for the 2%/2 mm gamma passing rates of PD and ArcCHECK was moderate, for prostate with lymphatic drainage and for the rectum plans it was weak.

**FIGURE 4:**
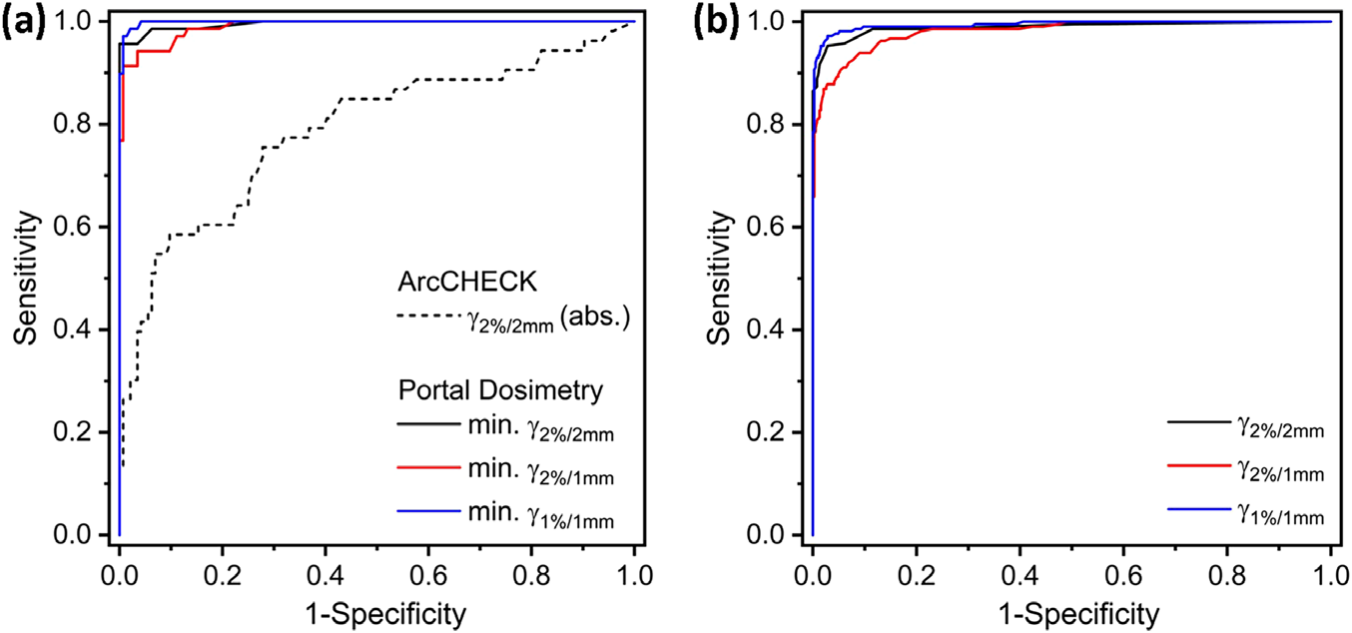
ROC curves for the QA tool comparison. The investigated plan acceptance criteria include γ_2%/2 mm_, γ_2%/1 mm_, and γ_1%/1 mm_ for PD and γ_3%/3 mm_, γ_3%/2 mm_, and γ_2%/2 mm_ for ArcCHECK gamma analysis. (a) ROC analysis per treatment plan. While ArcCHECK measurements were evaluated for the entire plan, for PD the worst arc's gamma passing rate was assigned to each plan. (b) ROC analysis for PD measurements analyzed per treatment field. Higher diagnostic performance is indicated by curves with larger area under the ROC curve.

An objective assessment of each individual QA criterion in terms of the capability to detect errors in the presented ensemble of clinical VMAT plans and the associated plan variants was done using the ROC methodology, with the obtained curves presented in Figure 4. Clearly, inaccuracies in VMAT plan delivery were detected more sensitive and more specific with the PD system than with ArcCHECK phantom measurements analyzed using the common gamma criteria γ_3%/3 mm_, γ_3%/2 mm_, and γ_2%/2 mm_ for the ArcCHECK phantom (Figure 4a). This is evident from AUC values of 0.995 ± 0.004 for PD 2%/2 mm gamma criterion, which is much closer to the optimum of 1 than the respective value of 0.818 ± 0.038 for the ArcCHECK. As summarized in Table 2, the AUC values of all investigated criteria are close to 1. Choosing suitable error detection thresholds enables an accurate detection of poor plan delivery. For PD optimum error detection thresholds, obtained as described in Section 2.7, were identified as 99.0%, 92.0%, and 86.0% for the criteria γ_2%/2 mm_, γ_2%/1 mm_, and γ_1%/1 mm_, respectively, when considering the minimum gamma passing rate for the plan's treatment fields (Table 2). If you look at all treatment fields individually, optimum error detection thresholds were identified as 99.6%, 94.5%, and 87.5% (Table 3). With regard to the ArcCHECK optimum thresholds of 99.3%, 98.4%, and 95.3% were obtained for γ_3%/3 mm_, γ_3%/2 mm_, and γ_2%/2 mm_. This choice simultaneously enables a very high sensitivity and specificity for spotting errors in treatment delivery.

**TABLE 2:** Error detection capabilities of various gamma criteria in PD and ArcCHECK measurements evaluated per plan

	Portal dosimetry			ArcCHECK		
QA criterion	γ_2%/2_ _mm_	γ_2%/1_ _mm_	γ_1%/1_ _mm_	γ_3%/3_ _mm_	γ_3%/2_ _mm_	γ_2%/2_ _mm_
AUC	0.995	0.990	0.997	0.848	0.823	0.818
Std. uncertainty AUC	0.004	0.006	0.002	0.034	0.037	0.038
Optimum threshold [%]	99.0	92.0	86.0	99.3	98.4	95.3

Besides the AUC and the corresponding standard uncertainty each criterion's optimum decision threshold is stated.

**TABLE 3:** Error detection capabilities of various gamma criteria in PD measurements evaluated per treatment field

	Portal dosimetry		
QA criterion	γ_2%/2_ _mm_	γ_2%/1_ _mm_	γ_1%/1_ _mm_
AUC	0.988	0.982	0.993
Std. uncertainty AUC	0.004	0.005	0.003
Optimum threshold [%]	99.6	94.5	87.5

Besides the AUC and the corresponding standard uncertainty each criterion's optimum decision threshold is stated.

### 3.3 Performance of portal dosimetry QA in clinical routine

**FIGURE 5:**
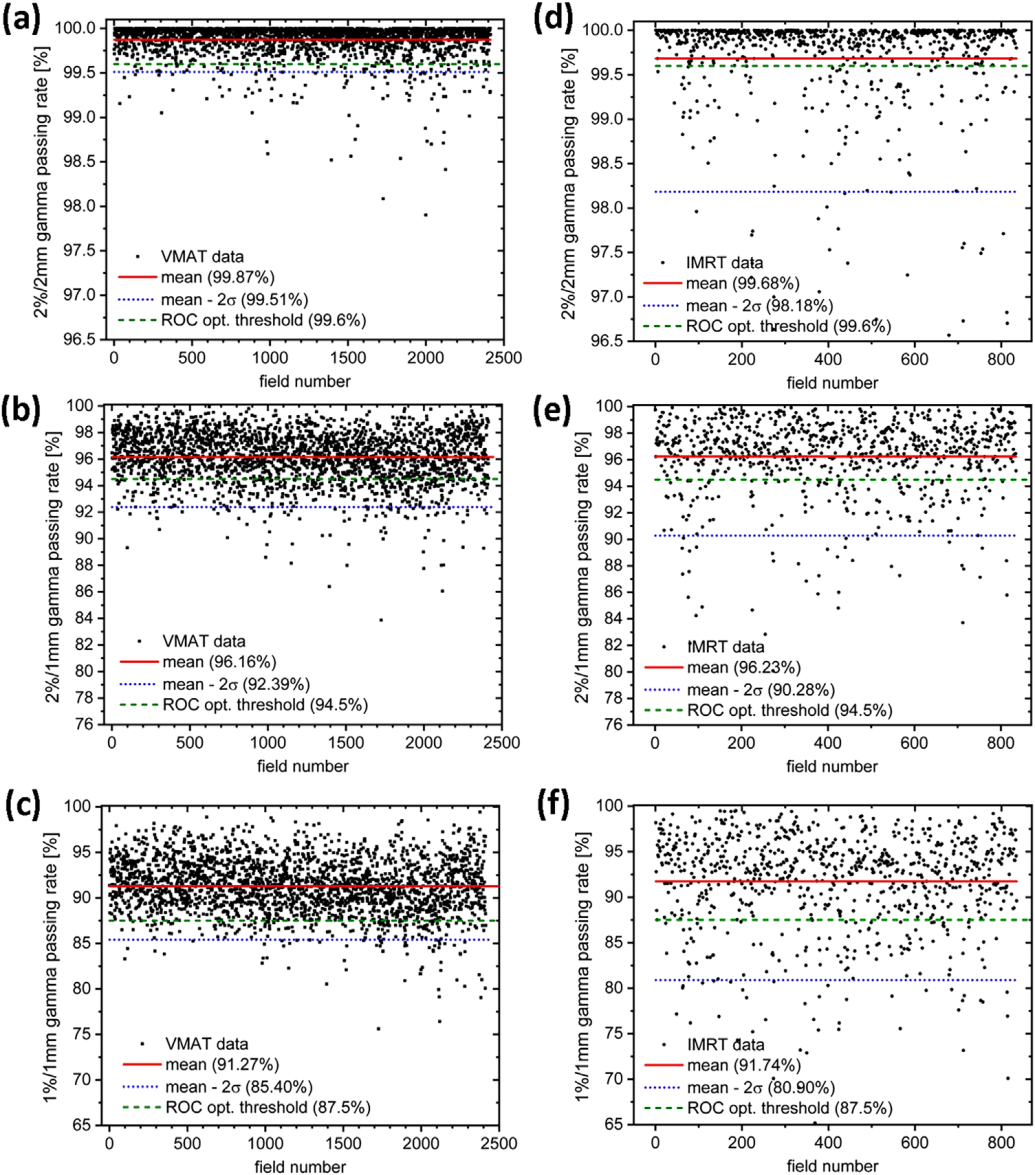
Control charts for the PD pre‐treatment QA gamma analyzes for VMAT on basis of a gamma criterion of (a) γ_2%/2 mm_, (b) γ_2%/1 mm_, and (c) γ_1%/1 mm_. Corresponding results for IMRT on basis of a gamma criterion of (d) γ_2%/2 mm_, (e) γ_2%/1 mm_, and (f) γ_1%/1 mm_. Black dots indicate the gamma passing rate obtained for each VMAT/IMRT treatment field individually, red solid lines indicate the criterion's overall mean, blue dotted lines the lower control limit (2σ) derived for the respective treatment technique, and green dashed lines the ROC derived optimum decision thresholds (Table 3).

Figure 5 displays a control chart‐like overview of all clinically recorded 1114 PD pre‐treatment QAs at the Halcyon after its installation and the previously described validation of the PD system for routine use, which covers a period of approximately two years.The majority of treatment plans (in total 943) utilized the VMAT delivery, while sliding‐window IMRT was clinically introduced at a later date. While the most common treatment sites for VMAT were head and neck (30% of investigated arcs) and prostate (28%), breast was the predominant site irradiated by IMRT (86% of investigated IMRT fields). The determined match of prediction and measurement depends on the irradiation technique which suggests a separate consideration of VMAT and IMRT.

Focusing on the 2%/2 mm gamma criterion (Figure 5a) its mean across the 2414 individual VMAT arcs is 99.87%, which nicely agrees with the corresponding mean obtained for the arcs considered for the evaluation of decision thresholds (Table 1). Compared to that the corresponding mean of 99.68% across the 835 IMRT treatment fields is slightly reduced (Figure 5d). An alternative tolerance threshold defined by the mean value minus two standard deviations σ results for VMAT delivery in a threshold value of 99.51% for the 2%/2 mm gamma passing rate, which is in good agreement with the value of 99.6% more rigorously obtained by means of an ROC analysis (see Table 3). Reasonable agreement is also evident for both, 2%/1 mm and 1%/1 mm gamma passing rates, from the comparison of Table 3 with the alternatively defined threshold values 92.4% (Figure 5b) and 85.4% (Figure 5c), respectively. As the passing rates for IMRT fields show larger variations, the mean value minus two standard deviations σ takes a reduced value of 98.18% for the 2%/2 mm gamma passing rate significantly deviating from the ROC‐derived optimum decision threshold. This is because no IMRT plans were included in the ROC analysis, which would be the prerequisite to derive adapted tolerance limits valid for IMRT. Only 6.2% (18.7%), 17.5% (19.9%), and 8.7% (17.1%) of the VMAT (IMRT) fields shown in Figure 5 fall below the optimum threshold from ROC for 2%/2 mm, 2%/1 mm, and 1%/1 mm gamma passing rates, respectively.

Regarding factors that cause failure, no correlation was found between the obtained gamma passing rates and the field's monitor units. For the dominant treatment sites, a site‐specific accumulation of fields deviating from the ROC thresholds was not observed. However, cervix treatments, which accounted for 2.2% of all VMAT arcs, showed a three‐fold increased failure probability with respect to the 2%/2 mm gamma criterion derived by ROC. This is attributed to the high plan complexity, which originates from the challenging competition of target volume coverage (partly including simultaneously integrated lymph node boosts) and OAR sparing.

## 4 DISCUSSION

All in all, the comparison of calibrated portal images acquired at the Halcyon with PD predictions in Eclipse demonstrated its reliability as a pre‐treatment QA tool. Its application is mainly anticipated to be the screening for potential errors in plan delivery. Two intrinsic aspects of PD are particularly advantageous in this context, firstly the fast data acquisition due to the much simplified measurement setup in comparison with many phantoms and secondly the automated workflow in the interplay with the dedicated PD module in Aria. At the same time, discarding any gantry angle dependent information while recording the integrated fluence of each VMAT arc and subsequent condensing of information to a single gamma passing rate leads to disadvantages related to the usage of PD. The main concern in this context is the appropriate response when a plan fails to comply with the established acceptance criteria, since its origin might be obscured. Therefore, when the obtained agreement of PD is unacceptable, preferably another dosimetry tool is required to judge how to proceed.

From the risk potential perspective it is essential to recognize the danger of missing real clinically significant errors, that is, false negatives, by concealing the effects of errors in the process of integrating and inevitably averaging out 3D data into a 2D plane. It is easy to create a case where a wrong fluence from one gantry angle is compensated by a wrong fluence from another. Therefore, it is necessary to use tight tolerances, to check the performance of PD for new plan types by means of an independent QA tool and to perform sample measurements with an independent device. In our clinic, results of PD QA are supported by a random sample survey of treatment plans additionally measured each week with the ArcCHECK phantom.

It should be noted, that the PD method is not invalidated by the aforementioned limitation, not least since VMAT delivery, particularly with multiple arcs, can also average out any errors in the final 3D dose distribution to the patient or recorded by a phantom, even though this averaging differs in way and extent between a 3D volume and a 2D plane. Therefore, the combination of a high agreement between predicted and measured portal doses, the proven high sensitivity to errors and the support by an independent verification method justify inferring a high delivery accuracy from good PD results.

Comparison of measurement systems, which rely on different physical measurement and data acquisition principles, requires great caution. As utilized in the presented study, PD and ArcCHECK measure different quantities in a different manner, use a different reference and are therefore by no means like for like methods. With its dense detector spacing, PD allows for the high resolution acquisition of the accumulated fluence only. QA with the ArcCHECK involves a different calculation engine, measures dose to a phantom, and is limited in resolution by a relatively large detector spacing, which comes with the risk of missing spots. Hence, the absence of a correlation between the respective results for clinical patient plans (Figure 2b), is not a surprise as one merely correlates uncertainties of both QA systems. As errors are introduced, both systems in general experience falling passing rates (Figure 3). However, the impact of certain errors on the QA outcome can be differently strong for different measurement principles utilizing different signal averaging. This explains the different correlation levels, which range from weak to moderate, for the different site‐specific plan modifications shown in Figure 3b.

The present study is based on a combination of a representative set of clinical VMAT plans and a selection of modified plans simulating erroneous plan delivery. By means of ROC analysis—a statistical performance measurement tool—reliable conclusions can be drawn with regard to the sensitivity and specificity of PD for pre‐treatment QA. Both quantities approach the ideal value of 1, as evident in Figure 4. The best performance is supposed to be achieved by the 1%/1 mm gamma criterion (Table 3), as evident from the highest AUC value. Focusing on a 2%/2 mm gamma criterion, PD promises ideal capabilities to detect errors using a threshold of 99.5%.

The implementation of pre‐treatment QA at the Halcyon was previously studied using multiple detector platforms, among others PD, by Laugeman et al.^38^ This work used a comparatively smaller number of 56 treatment plans for validation of plan generation and delivery. In contrast to our work the reported tolerance and action limits in their work were not the result of a direct investigation of the efficiency to detect erroneous treatment delivery, but derived utilizing equations from statistical process control.^39^, ^40^, ^41^ They limited the evaluation to 3%/2 mm gamma passing rates, as recommended by TG‐218,^6^ and for VMAT yielded a mean passing rate of 99.9% with tolerance and action limits of 99.5% and 98.4%, respectively. In our study, the relaxed dose requirements of a γ_3%/2 mm_ criterion seemed less suitable to us as it expectedly is less sensitive given the overall excellent agreement observed for PD. Instead, our results for the mean 2%/2 mm gamma passing rate of 99.9% and the optimum decision threshold of 99.5% compare well with the above stated values for γ_3%/2 mm_.^38^

Previous work by Gay et al.^42^ also examined the detectability of MLC positioning errors on a preclinical Halcyon linac and concluded that systematic errors are likely to be detected by PD, whereas small random errors are less likely to be detected. While similar in approach, our work adds a sophisticated derivation of decision thresholds for optimum error detection capabilities by means of an ROC analysis. Sticking to these thresholds almost perfect sensitivity and specificity are achieved, even for plans with simulated random errors of only small dosimetric impact. In addition, our data for PD is supported by the evaluation of corresponding measurements with the ArcCHECK phantom, which is widely recognized for its suitability to efficiently detect errors in plan delivery.^37^ The observation of a reduced sensitivity to detect delivery errors in ArcCHECK measurement is supposed to be mainly due to the reduced pixel density of about 1 versus 30 pixels/cm. A non‐perfect phantom modelling in the TPS might also contribute to the weaker performance of the ArcCHECK.

There are some intrinsic limitations to the use of PD as QA tool. Calculation of portal doses requires a separate portal dose image prediction algorithm instead of the actual Acuros XB patient dose calculation algorithm. Inaccuracies in the latter occurring downstream from fluence calculation may thus be masked.^11^ Although widely in use in plan‐specific QA, the gamma analysis is known to possess limitations.^43^ Furthermore, the measurements themselves can suffer from digital detector issues, for example electronic noise or detector overload, and deviations in the dosimetric calibration of the imager panel.

While the preconfigured nature of the Halcyon system introduces some consistency across clinics, the adoption of the presented tolerance limits additionally requires the plan optimization process and the resulting plan complexity to be comparable. A multicentric study would give insight into limits of inter‐clinic transferability of universal thresholds for plan‐specific QA. Moreover, this study solely concentrated on VMAT plans at the Halcyon, while IMRT plans were not the focus. Therefore, the obtained results of this work cannot directly be applied to the IMRT pre‐treatment QA, which requires a separate validation.

As PD proved valuable for plan‐specific pre‐treatment QA, we plan to approach the retrospective evaluation of daily in‐vivo PD. At the Halcyon linac transit images of each radiation field are automatically recorded for each daily treatment by its EPID. These exit images are affected by internal or external anatomy (e.g., organ motion or filling, body weight), and potentially can indicate clinical errors (e.g., insufficient patient positioning) in the course of treatment or situations benefitting from adaptive radiation therapy. Therefore, complementing the sophisticated analysis of daily CBCTs the evaluation of daily in vivo PD promises to be a simple, feasible method for treatment monitoring.

## 5 CONCLUSION

Varian PD is an integrated, fast and precise plan‐specific pre‐treatment QA solution, which can reliably be used to screen for errors in VMAT delivery at the Halcyon linac. The portal dose prediction for the clinical patient plans proved to very accurately simulate the EPID measurements with an average gamma passing rate of γ_2%/2 mm_ = 99.90%. Both simulated systematic and random errors in treatment delivery were detected with almost perfect sensitivity and specificity using the ROC optimized decision thresholds. In our clinic, PD QA results will be supported by a random sample survey of treatment plans additionally measured each week with the ArcCHECK phantom.

## AUTHOR CONTRIBUTIONS

Gary Razinskas, Otto A. Sauer, and Sonja Wegener designed the study. Gary Razinskas obtained data and carried out the data analysis with Robert Schindhelm. All authors discussed the results. Gary Razinskas wrote the first draft of the manuscript. Robert Schindhelm, Otto A. Sauer, and Sonja Wegener revised the manuscript. All authors agree with the submission.

## CONFLICT OF INTEREST STATEMENT

There are no potential conflicts of interest to disclose.
